# Are There Differences in the Homogeneity of the Parts of Tablets Obtained after Subdivision?—A Preliminary Assessment Using an X-ray Microtomography

**DOI:** 10.3390/pharmaceutics14091850

**Published:** 2022-09-01

**Authors:** Michał Meisner, Piotr Kuśnierz, Piotr Duda, Sławomir Wilczyński, Beata Sarecka-Hujar

**Affiliations:** 1 Department of Basic Biomedical Science, Faculty of Pharmaceutical Sciences in Sosnowiec, Medical University of Silesia in Katowice, Kasztanowa Str. 3, 41-200 Sosnowiec, Poland; 2 Institute of Biomedical Engineering, Faculty o Faculty of Science and Technology, University of Silesia, 41-200 Sosnowiec, Poland

**Keywords:** subdivision, tablets, solid dosage forms, microtomography

## Abstract

*Aim:* The study aimed to analyze the weight and homogeneity of the parts of tablets containing carbamazepine and tablets with trazodone hydrochloride, obtained after subdivision with a kitchen knife. X-ray microtomography was used for homogeneity analysis. *Methods:* 30 tablets with carbamazepine and 30 tablets with trazodone hydrochloride were analyzed in terms of weight uniformity after subdivision. Then, seven tablets of each type were analyzed using an X-ray microtomography (Phoenix vǀtomeǀx, General Electric). The absorption of X-rays by an object is proportional to its density. In turn, measurement of the density of the analyzed object in a microtomographic image is the grayscale level. Based on the correlation between the grayscale value and the reference density, from the calibration phantom, we were able to determine the density of any area of the tablet’s scan. *Results:* During the subdivision, the weight loss exceeded 3% for two carbamazepine tablets, while for trazodone tablets, none lost more than 3%, which is the limit recommended by Food and Drug Administration (FDA). As to the density of the tablets resulting from the microtomographic analysis, two of the whole tablets containing trazodone hydrochloride had a significantly higher density than the remainder (*p* < 0.001). Similarly, some differences in density were observed in the analysis of the density of tablets of carbamazepine (*p* = 0.008). Parts of one of the analyzed tablets with trazodone obtained after subdivision differed in terms of pixel brightness, thus density. On the other hand, the uniform density was observed for parts of the split tablets containing carbamazepine. *Conclusions:* Parts of the trazodone hydrochloride tablets obtained after subdivision differed in terms of homogeneity and weight. Microtomographic methods may be an interesting and useful method for evaluating the uniformity of compounds in solid dosage forms.

## 1. Introduction

Available data show that the phenomenon of dividing tablets is common all over the world. The reasons for the popularity of this practice are factors important not only for the patient (convenience, price) but also for the doctors (the possibility of obtaining the optimal dose for a specific patient, avoiding side effects). Therefore, tablets are subdivided not only by patients at home but also by qualified personnel in medical facilities [1,2]. The division of tablets into parts allows for and facilitates the gradual increase of doses of drugs during the implementation of pharmacotherapy for many diseases [3,4]. The study by Gracia-Vásquez et al. [5] demonstrated that over 80% of analyzed patients subdivided their tablets, and it was performed mostly due to difficulties with swallowing the whole tablets. In addition, the authors observed that patients split the tablets mostly with a knife, and only 10% of them used a special pharmacy cutter for this purpose. At the same time, 72.5% of respondents were aware of the fact that not all tablets can be broken [5].

In order to meet the general expectations, many pharmaceutical companies have started the production of tablets with special dividing lines. However, the fact that the manufacturer places a score line on a tablet does not always mean that a given preparation should be divided. The score line often only helps to divide the tablet into smaller parts, which in turn improves the administration of the drug to the patient but does not guarantee 100% equal doses of the drug in the parts obtained. This can lead to various adverse effects. Thus, to facilitate tablet administration, the manufacturer only needs to produce a tablet that can be divided (without mass loss) but does not have to prove a homogeneous spread of active pharmaceutical ingredient (API) distribution.

Despite the apparent simplicity of this operation, the methodology and effects of the tablet subdivision are still being studied. The homogeneous distribution of API in the tablet is a crucial factor for obtaining the same doses in all parts of the divided tablet. Uneven division of the tablet may cause significant fluctuations in the administered dose [6,7]. Therefore, the clinical significance of the potentially unequal subdivision of tablets containing potent substances or those with a narrow therapeutic index should be taken into account. In general, splitting tablets is not recommended; it is permissible to split medicines where the dose is not a factor in determining the effectiveness of the medicine, e.g., vitamins or painkillers [8]. In turn, according to European regulators, the practice of dividing tablets is discouraged regardless of the type of the drug to be divided [9]. However, the European Pharmacopoeia (Ph. Eur.) and the FDA provide some information based on which it is possible to define parameters that should be controlled for divided tablets, i.e., mass uniformity, uniform composition, and disintegration time as for undivided tablets. The FDA also states that the weight loss after subdivision should not exceed 3% [10,11].

Previously, it was suggested that modern imaging methods such as X-ray microtomography, optical coherence tomography (OCT), and terahertz imaging can be useful for checking the homogeneity of the portion of tablets obtained after subdivision [12]. Computer X-ray microtomography is a radiographic imaging technique that allows non-invasive characterization of the microstructure of the analyzed samples. With this technique, it is possible to accurately determine the three-dimensional structure of opaque objects at a very high resolution [13].

The aim of the study was to analyze parts of the tablets containing carbamazepine and trazodone hydrochloride, obtained after subdivision, in terms of their mass as well as their homogeneity assessed based on microtomographic scans.

## 2. Materials and Methods

### 2.1. Tablets Selected for Analysis

In the present study, tablets containing trazodone hydrochloride (Trittico CR; Aziende Chimiche Riunite Angelini Francesco A.C.R.A.F. S.p.A., Rome, Italy) and tablets containing carbamazepine (Finlepsin 200 retard; Teva Pharmaceuticals Polska Sp. z o. o., Warsaw, Poland) were analyzed. According to the manufacturer’s recommendation, the carbamazepine tablets may be subdivided into two equal parts. In turn, the trazodone hydrochloride tablets have two score lines which allow the division of the tablet into three equal parts according to the manufacturer’s recommendation (Table 1).

### 2.2. Evaluation of the Whole Tablets

Tablets selected for the study were assessed following the requirements of the Polish Pharmacopoeia XI (FPXI) for this drug form in terms of the appearance of the tablets (with the use of a magnifier), the size measurement (i.e., the diameter, thickness, length and width of any selected 5 tablets were measured using caliper with an accuracy of 0.1 mm), and the weight uniformity (*n* = 20 randomly selected tablets of one series were weighed and the arithmetic mean of tablets weight was determined). For weighting, the same analytical balance (Radwag, Radom, Poland), with analytical accuracy of 0.1 mg was used.

### 2.3. Evaluation of Parts of the Tablets

Thirty whole tablets of analyzed type, randomly selected from one batch were individually weighed before subdivision. Then, tablets were subdivided using a kitchen knife (the most common way to split tablets) by one of the authors and a subsequent procedure of weight analysis was performed following both the Ph. Eur. and the United States Pharmacopeia (USP) as there are some discrepancies between the two regulators [9].

According to the Ph. Eur., each tablet is subdivided and only one of its parts is weighed. Each fraction must be between 85 and 115% of the average mass of these 30 weightings. Only one part out of the 30 may be outside this limit. The tablets failed the test if more than one individual mass was outside the range of 85–115% of the average mass, or if any individual mass was outside the range of 75–125% of the average mass [9,14].

According to USP, 10 out of the 30 tablets were individually weighed. Then, each tablet was divided, resulting in 20 fragments which were weighed. The number of tablet parts outside the 85–115% range of average mass, as well as the number of tablet parts outside the 75–125% range of average mass, was counted. In addition, the relative standard deviation (RSD) of the half-tablet weights was calculated using the following formula:

$$\mathrm{RSD}=\frac{\mathrm{SD}\;\mathrm{for}\;\mathrm{measured}\;\mathrm{variable}}{M\;\mathrm{of}\;\mathrm{measured}\;\mathrm{variable}\;}*100$$

where RSD—relative standard deviation; SD—standard deviation; M—mean.

The product passed the USP uniformity test if only one tablet part was outside the 85–115% range (but within the 75–125% range) and if the RSD was 6% or less [9].

Mass loss after subdivision was calculated as the difference between the mass of the whole tablet and the sum of the masses of the tablet’s parts according to the equation:

Mass loss  [g]=mass of the whole tablet [g]−(mass of part one [g]+mass of part two [g])

### 2.4. Microtomographic Analysis

Scans for each tablet were registered using X-ray microtomography (Phoenix vǀtomeǀx, GE Sensing & Inspection Technologies GmbH, Wunstorf, Germany).

**Figure 1:**
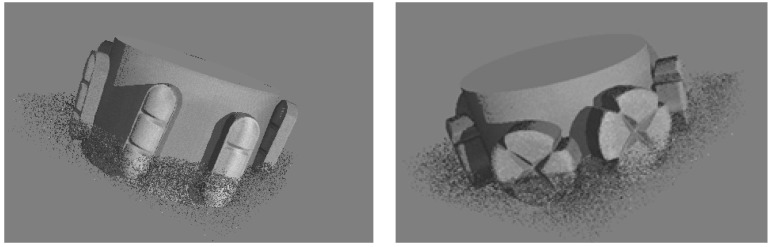
The way of arranging the tablets around the microtomography cylinder (trazodone hydrochloride tablets on the **left**, carbamazepine tablets on the **right**).

The tablets were placed on polymer support and then they were scanned at an energy of 140 kV. X-rays passing through the sample were converted into visible radiation using a YAG: Ce scintillator. This allowed for image recording with a resolution of 2024 × 2024 pixels. One thousand eight hundred scans were recorded for each tablet with a total scan time of 45 min and an amperage of 145 μA. To obtain the maximum image resolution—the smallest possible voxel—the appropriate distance between the sample and the matrix was established. The distance of the tablet scanned in the microtomography from the matrix is limited by its rotation, because the entire rotating tablet must match the detector matrix (Figure 1). Projections were acquired every 0.4° with a total object rotation of 180°. To improve the signal-to-noise ratio, the measurements from every three acquisitions made at each tablet rotation step were averaged. The parameters determined in this way allowed to register an image with optimal contrast with a resolution of 10 μm. The scanner manufacturer’s software, Phoenix Datos|x 2.0 CT^®^ software (GE Sensing & Inspection Technologies GmbH, Wunstorf, Germany), was used for image acquisition and image reconstruction.

**Figure 2:**
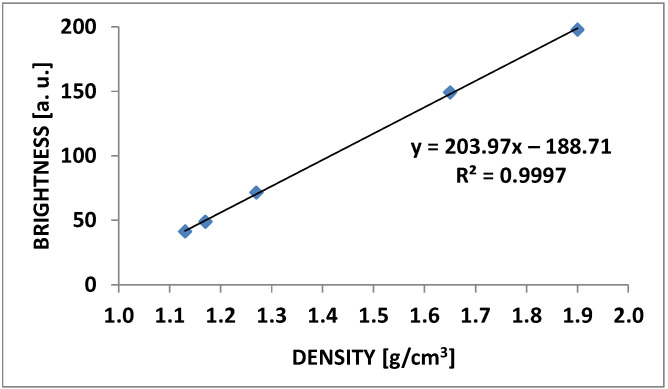
The correlation between the brightness of the pixels and the density of the composition was assessed in the analysis of the calibration phantom.

The absorption of X-rays by an object is proportional to its density. In turn, the measure of the density of the analyzed object in a microtomographic image is the grayscale level. The Micro-CT HA Phantom D32 calibration phantom was also scanned under the same conditions as the analyzed tablets to establish the grayscale level of reference density (W1—1.13 g/cm^3^; W2—1.16 g/cm^3^; W3—1.26 g/cm^3^; W4—1.65 g/cm^3^; W5—1.90 g/cm^3^). In the adopted research model, “bright” pixels represent high-density areas, while “dark” pixels represent low-density areas. Based on the histograms obtained with the ImageJ software (ImageJ 1.53a; National Institutes of Health, Bethesda, MD, USA), a calibration curve was determined. This allows for determining the density of any area of the tablet scan (Figure 2).

### 2.5. Statistical Analyses

The data were analyzed using the STATISTICA 13.0 software. Quantitative variables (weight, brightness, and tablet density) were analyzed as mean values (M) with standard deviation (SD) and medians with minimum–maximum values. The normality of the distribution of quantitative data was evaluated by the Shapiro–Wilk W test. For the homogeneity of the variance, Levene’s test was used. Comparisons of the mean values of quantitative data among all analyzed subgroups were performed with the use of the following tests: analysis of variance (ANOVA), when the distributions of data do not differ from the normal distribution, or the Kruskal–Wallis test, when the distributions of quantitative data differ from a normal distribution or the assumption of homogeneity was violated. In the case of significant differences observed using the Kruskal–Wallis test, the Mann–Whitney U test was used for post hoc pairwise comparisons.

## 3. Results

### 3.1. General Characteristics of the Analyzed Tablets

The carbamazepine tablets were white to yellowish, round, flat, and with rounded edges. The trazodone hydrochloride tablets were of oblong shape, with rounded edges. The dimensions of the tablets met the FPXI requirements (Table 2).

### 3.2. Weight Analysis before and after Subdivision

The whole analyzed tablets containing carbamazepine or trazodone hydrochloride showed weight uniformity and were fully compliant with pharmacopoeial standards. No weight of the whole tablets containing trazodone hydrochloride exceeded the permitted variation of ±7.5% as well as no weight of the whole tablets containing carbamazepine exceeded the permitted variation of ±5%.

Table 3 presents compliance to regulatory requirements of divided tablets with both carbamazepine and trazodone hydrochloride according to Ph. Eur. and USP. Both analyzed types of tables passed the uniformity test performed following Ph. Eur. Guideline; however, tablets containing trazodone hydrochloride failed the test following the USP guideline (Table 3).

Mean values of the mass of whole tablets containing carbamazepine and trazodone hydrochloride as well as the mass of parts of the tablets obtained after subdivision are shown in Table 4.

The weights of the parts of the tablets after division and their theoretical weight were significantly different from each other. In the post hoc analysis, the weights of the right and left parts of the trazodone hydrochloride tablets were comparable (*p* = 0.091). As middle parts of the trazodone hydrochloride tablets had the highest weight, weights of both the right and left parts differed significantly from the mass of the middle parts (*p* < 0.001 each). Comparing the obtained mass of parts of tablets and theoretical mass showed significant differences in all comparisons (*p* < 0.001). In the case of carbamazepine tablets, the weights of the left parts differed significantly from the mass of the right parts of the tablets (*p* = 0.006). The weights of the right parts of carbamazepine tablets differed significantly from the theoretical weights (*p* = 0.036) while the left parts of carbamazepine tablets did not differ from the theoretical mass of the tablets (*p* = 0.473). Data of mass loss are shown in Table 5. During the subdivision, the weight loss of two tablets with carbamazepine exceeded 3% (i.e., 3.33% and 4.43%, respectively) while for trazodone hydrochloride tablets, none lost more than 3%.

### 3.3. Microtomographic Analysis

**Figure 3:**
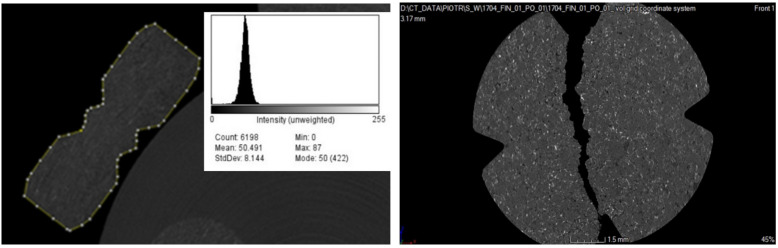
Exemplary microtomographic scans for the whole carbamazepine tablet as well as for the halves of the tablet.

The microtomographic scans of the tablets analyzed in the present study are shown in Figure 3. We analyzed seven tablets containing trazodone hydrochloride and seven tablets containing carbamazepine. For each whole tablet, 20 random scans (out of 1800) were analyzed using imaging methods.

**Figure 4:**
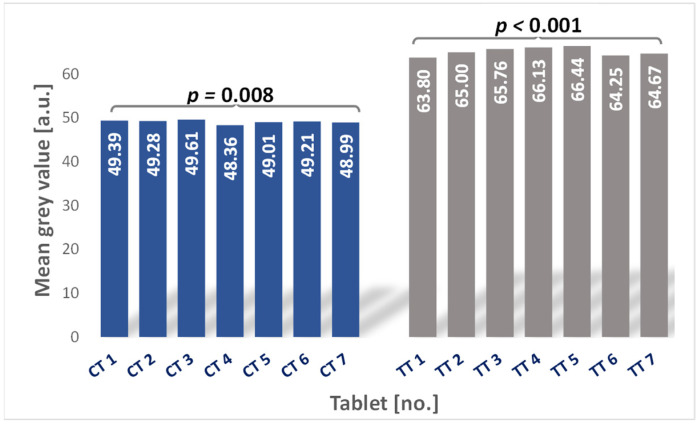
The mean greyscale value of the pixels (from *n* = 20 scans of each whole tablet) corresponds to the density of the composition. CT-carbamazepine tablet; TT-trazodone hydrochloride tablet.

In the case of the tablets containing trazodone hydrochloride, two of the whole tablets have a significantly higher density than remained (*p* < 0.001). Similarly, significant differences were observed in the analysis of the density of the whole tablets containing carbamazepine (*p* = 0.008) (Figure 4).

**Figure 5:**
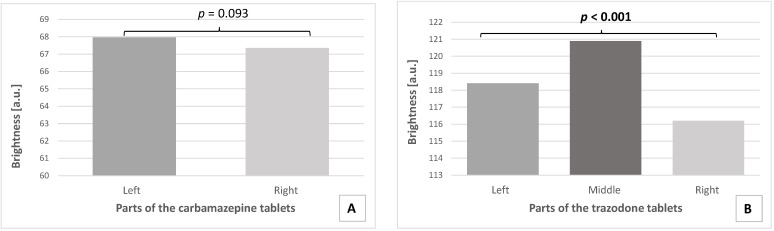
Brightness distribution of the obtained parts of the carbamazepine tablets (**A**) and trazodone hydrochloride tablets (**B**). Significant difference is in bold.

The mean brightness of the 20 scans of the right and left parts of carbamazepine tablets were read from the obtained histograms. Similarly, 20 scans of the right, middle, and left parts of the trazodone tablets were analyzed in terms of brightness. The distribution of the mean values of the pixel brightness of the tested tablets is presented in Figure 5.

The microtomographic analysis of the divided carbamazepine tablets showed that the mean brightness of the left part of the tablets (67.96 ± 1.26) was comparable to the brightness of the right part of the tablets (67.35 ± 1.47). Therefore, uniform density for parts of the tablets containing carbamazepine after the division was observed (*p* = 0.093) (Figure 5A).

Parts of one of the analyzed tablets with trazodone hydrochloride obtained after subdivision differed in terms of pixel brightness, thus density (*p* < 0.001). For this preparation, the middle part of the tablet with trazodone hydrochloride was significantly brighter (120.90 ± 2.67) than both the left part (118.41 ± 1.38) and the right part (116.20 ± 3.17) of the tablet. In addition, the left part was significantly brighter than the right part (Figure 5B).

## 4. Discussion

In the present study, we observed that the weights of the carbamazepine and trazodone hydrochloride parts of tablets, obtained after subdivision with a kitchen knife, differed significantly from each other. The difference was especially significant for the trazodone hydrochloride tablets because their middle part was heavier than remained parts. Commonly, the clinical practice requires the division of the tablet into two equal parts however trazodone hydrochloride tablets may be divided into three parts. In the study by Starling et al. [15] tablets with trazodone hydrochloride were the only tablets out of 20 analyzed types of tablets which can be divided into three parts by geriatric patients. We observed that mass loss for only two tablets with carbamazepine exceeded 3% (mean mass loss was 0.9%) while none of the trazodone hydrochloride tablets lost more than 3% (mean mass loss was 0.5%). Obtained results are comparable with the results presented by Teixeira et al. [16]. The carbamazepine tablets analyzed by authors showed a suitable behavior in terms of their ability to undergo subdivision, with mass loss close to 0%. The greater mass loss (0.2 ± 0.3%) was found in the case of trazodone tablets when the tablets were divided with a score line [16]. The authors demonstrated also that it is hard to divide trazodone tablets into equal halves (i.e., out of score line) but the hardness of the trazodone tablets divided into two or three parts was comparable [16]. Trazodone hydrochloride tablets analyzed in our study were oblong and such a shape was found to give better results in the division compared to a round shape [17].

In our study, we assumed that microtomographic analysis of tablet parts after subdivision may help in assessing their homogeneity. A microtomographic method is one of the modern techniques used for more accurate analysis of pharmaceutical formulations, especially solid dosage forms in terms of API distribution. Homogeneity of the tablet halves is one of the most important factors affecting the correctness of the subdivision of the tablets if uniform doses that are to be obtained after this process. It is especially important when a multiple-unit particulate system is subdivided. Failures in tablet uniformity may, in consequence, lead to drug content variations of up to 10% in tablet halves putting doubt in the safety of splitting this class of tablets [18,19].

In the case of microtomographic analysis of the whole tablets, we observed some differences first in greyscale value and, in consequence, in density between the individual tablets both for carbamazepine tablets as well as for trazodone hydrochloride tablets. However, in the case of homogeneity of the tablet fragments after subdivision, the significant differences in density were observed only for the trazodone hydrochloride tablets. Due to these results, we may assume that dividing the prolonged-release tablets containing 75 mg trazodone hydrochloride does not guarantee obtaining parts with the same amount of the active substance. This, in turn, may cause a risk of fluctuations in the dose of medication taken by the patient. Our study, however, did not aim to analyze clinical effects. Therefore, it would be necessary to consult doctors whether the differences shown will affect the effectiveness of the treatment if only one third of the given tablet is used.

However, data on adapting this technique to analyze API distribution in the solid dosage forms are scares. Previously, a study by Wray et al. [20] evaluated X-ray tomography to analyze the caffeine distribution as a technique complementary to imaging with Fourier Transform Infrared (FTIR) spectroscopy. It was demonstrated that tomography may be used in conjunction with FTIR imaging to produce a density map of the tablets [20]. The microstructure of the tablets containing ibuprofen was also assessed with microtomography by Schomberg et al. [13]. The authors showed that clusters of the active substance are present for all the tablets which contained >1 wt% of ibuprofen.

Tablets in the present study were split into parts using a kitchen knife and this dividing method is one of the most commonly used by patients [5]. However, data indicate that the best method to divide tablets is the one with a special pharmacy cutter since the smallest weight loss of the tablets is then observed. Proper placement of the tablet into such a guillotine is nevertheless necessary. Data demonstrated that the loss of mass is greater if the tablet is divided by hand [21]. Divided tablets also show greater friability compared to undivided tablets, but tablets divided by hand are more fragile than those divided by a cutter [19]. In a study by Gharaibeh and Tahaineh [22], the tablet cutter was found to be a better way to subdivide tablets than the other methods, i.e., by hand or with a knife. The tablet halves from the pharmacy cutter passed the weight uniformity test for most of the tablets analyzed by the authors [22].

Prolonged-release tablets that can be subdivided for dose titration should maintain a controlled-release profile in proportion to the reduced dose. Thus, such drug forms must have appropriate mechanical properties for division, i.e., no crushing and the production of the parts with the same dose as each other [16].

## 5. Conclusions

The results of the present preliminary study suggest that there might be differences in the distribution of trazodone hydrochloride in the parts of the tablets obtained after subdivision as we observed differences in the density of these parts. No such differences were found in the case of carbamazepine tablets. X-ray microtomography supported by techniques of image analysis and processing helps to analyze the internal structure of pharmaceutical product and may be a useful method for evaluating the homogeneity of the compounds in the tablets that undergo a subdivision process. Thus, the methodology may be a part of the development process before marketing approval. Although the study is one of the few studies showing microtomographic scans in the analysis of pharmaceutical products, further research on the topic is needed.

## Figures and Tables

**Table 1:** Characteristics of the selected tablets.

Preparation	Active Pharmaceutical Substance; Dose	Manufacturer’s Recommendation on Subdivision	Score Line	Number of Parts Obtained after Subdivision *
Trittico CR	trazodone hydrochloride; 75 mg	yes	yes	3
Finlepsin 200 retard	carbamazepine; 200 mg	yes	yes	2

* According to manufacturer’s recommendations.

**Table 2:** Characteristics of the selected drugs.

Preparation	Shape	Length of the Tablets [mm] M ± SD	Width of the Tablets [mm] M ± SD	The Thickness of the Tablets [mm] M ± SD	Diameter of the Tablets [mm] M ± SD
Trazodone hydrochloride tablets	oblong	12.05 ± 0.01	4.05 ± 0.04	3.03 ± 0.04	-
Carbamazepine tablets	round	-	-	3.47 ± 0.01	10.10 ± 0.01

M—mean; SD—standard deviation.

**Table 3:** Compliance with regulatory requirements of weights of subdivided tablets according to Ph. Eur. and USP.

Analyzed Tablets	Uniformity Test for Subdivided Tablet Acc. To Ph. Eur.			Uniformity Test for Subdivided Tablet Acc. To USP				
	Outside the Range of 85–115% of the Average Mass *N*	Outside the Range of 75–125% of the Average Mass *N*	Result of the Test	Number of Tablets	Outside the Range of 85–115% of the Average Mass *N*	Outside the Range of 75–125% of the Average Mass *N*	RSD [%]	Result of the Test
Carbamazepine tablets	0	0	Pass	10	0	0	5.75	Pass
Trazodone hydrochloride tablets	1	0	Pass	10	8	1	13.95	Fail

Ph. Eur.—European Pharmacopeia; USP—United States Pharmacopeia; RSD—relative standard deviation.

**Table 4:** The weight of the whole tablets and mass of the parts of the tablets obtained after subdivision.

	Before Subdivision	After Subdivision				
	Whole Tablets *N* = 30	Left Parts of the Tablets *N* = 30	Middle Parts of the Tablets *N* = 30	Right Parts of the Tablets *N* = 30	Mass of the Theoretical Parts of the Tablets	*p*
Trazodone hydrochloride tablets, M ± SD	0.142 ± 0.001	0.043 ± 0.002	0.054 ± 0.003	0.044 ± 0.002	0.047 ± 0.0005	<0.001 *
Carbamazepine tablets, M ± SD	0.301 ± 0.002	0.152 ± 0.008	-	0.146 ± 0.007	0.150 ± 0.001	0.015 **

M—mean; SD—standard deviation; * *p* from Kruskal–Wallis analysis; post hoc analysis: right part vs. left part of the trazodone hydrochloride tablets (*p* = 0.091); right part vs. middle part of the trazodone hydrochloride tablets (*p* < 0.001); left part vs. middle part of the trazodone hydrochloride tablets (*p* < 0.001); right or left or middle parts of tablets vs. theoretical mass (*p* < 0.001); ** *p* from Kruskal–Wallis analysis; post hoc analysis: left part vs. right part of the carbamazepine tablets (*p* = 0.006); right parts or left parts vs theoretical mass (*p* = 0.036 and *p* = 0.473, respectively). Significant differences are in bold.

**Table 5:** Mass loss after subdivision.

	Mass Loss [g]	Mass Loss [%]
Trazodone hydrochloride tablets, M ± SD	0.0008 ± 0.0005	0.544 ± 0.354
Carbamazepine tablets, M ± SD	0.003 ± 0.003	0.932 ± 0.941

M—mean; SD—standard deviation.

## Data Availability

The data presented in this study are available on request in the Department of Basic Biomedical Science, Faculty of Pharmaceutical Sciences, Medical University of Silesia in Katowice (Poland). The data are not publicly available due to privacy restrictions.
